# The Impact of Errors in Copy Number Variation Detection Algorithms on Association Results

**DOI:** 10.1371/journal.pone.0032396

**Published:** 2012-04-16

**Authors:** Nathan E. Wineinger, Hemant K. Tiwari

**Affiliations:** 1 Section on Statistical Genetics, Department of Biostatistics, University of Alabama, Birmingham, Alabama, United States of America; 2 Scripps Translational Science Institute, Scripps Health, La Jolla, California, United States of America; University of Wuerzburg, Germany

## Abstract

The inaccuracy of copy number variation (CNV) detection on single nucleotide polymorphism (SNP) arrays has recently been brought to attention. Such high error rates will undoubtedly have ramifications on downstream association testing. We examined this effect for a wide range of scenarios and found a noticeable decrease in power for error rates typical of CNV calling algorithms. We compared power using CNV calls to the log relative ratio and found the latter to be superior when error rates are moderate to large or when the CNV size is small. It is our recommendation that CNV researchers use intensity measurements as an alternative to CNV calls in these scenarios.

## Introduction

Copy number variation (CNV) is a significantly large portion of overall human genetic variation that may influence numerous diseases and traits. CNVs have been found to be associated with autism [1], autoimmune disease [2], HIV transmission [3], obesity [4], and schizophrenia [5] among others. CNVs have been hypothesized to contribute to the missing heritability left unexplained by single nucleotide polymorphisms (SNPs) [6], [7]. Despite the large fraction of the human genome that CNVs encompass, genome-wide association studies based on complex traits and CNVs have been largely unsuccessful compared to SNP counterparts. Some have even argued that tests based on CNVs may be unnecessary as common CNVs of disease relevance are likely well-tagged by neighboring SNPs [8].

There are numerous possibilities why tests based on CNVs have been less successful than what was once predicted. Many CNVs are individually rare, and are thus poorly designed for traditional tests of association. Methods such as those based on the overall burden of CNV have been proposed [9], but are likely too imprecise to be used universally. However, a more concerning issue is that the identification of CNVs on a genome-wide level, usually based on CNV calling algorithms, often contains numerous errors. Zhang *et al*. (2011) [10] recently examined the ability of popular CNV calling algorithms to detect known CNVs from data on the Affymetrix 6.0 array. Among their findings, they found small CNVs and common CNVs have very low recovery rates (i.e. high rate of false negatives), which no doubt will affect the results of association tests.

While the impact of SNP genotyping errors on association analyses has been extensively studied [11], [12], a thorough examination of CNVs has not been done despite there being marked differences in testing procedures and errors between SNPs and CNVs. We examined the impact of errors in CNV calling algorithms on association testing via simulation, and compared the results to those based on intensity measurements in the form of the log relative ratio (LRR). We find the LRR to be superior to CNV calling when the data is wrought with calling errors and when CNV sizes are small. It is our recommendation, until the accuracy of CNV calling algorithms improve substantially, that researchers use functions of the overall copy number intensities to test of trait associations.

## Methods

Typical hidden Markov model (HMM) based CNV calling algorithms, such as PennCNV [13] or the Birdseye application within Birdsuite [14], assign integer copy number states to segments of DNA based on hybridization intensities and software parameters with state space ranging from zero to four. Any genetic locus that contains a single copy duplication or deletion on one parental chromosome can be appropriately modeled by these algorithms. For example, a deletion on both pairs of parental chromosomes would have a copy number of zero, while a single duplication on both pairs of parental chromosomes would have a copy number of four. Because of the state space limitation, these algorithms can only differentiate copy number states within its range – unable to properly model non-integer values resulting from heterogeneous copy number or regions that include greater than four states.

Due to this restriction we only consider CNVs with maximum copy number less than or equal to four. We define X as the true, but unobserved integer copy number state at a given locus, such that the sample space of X is {0, 1, 2, 3, 4} with frequencies defined in Table S1. In practice, X is rarely estimated without uncertainty. Often specialized laboratory techniques, such as quantitative PCR, need to be employed to accurately assess the existence and boundaries of CNVs. Even so, it is impractical to perform these tests across the genome for many samples. CNV researchers are often left using the results from CNV calling algorithms to perform genome-wide CNV association analyses. However, studies have shown these algorithms are prone to errors which may have a profound effect on power [10], [15], [16].

### Errors in CNV Calling Algorithms

We focus on two types of errors that can occur within typical HMM-based calling algorithms: incorrectly assigning a non-reference copy number to a sequence that truly is the reference copy number; and incorrectly assigning the reference copy number to a sequence that truly is not the reference copy number. We refer to these errors as false positives and false negatives, respectively. Although not always the case, we assume copy number of two represents the reference copy number state. Therefore the false positive error rate (ν_p_) is the frequency at which a copy number state other than two is assigned to a region present in two copies; and the false negative error rate (ν_n_) is the frequency at which a copy number state of two is assigned to a region present in X  =  0, 1, 3, or 4 copies (2 being normal). Zhang *et al*. (2011) [10] call one minus ν_n_ the recovery rate and show it can be very low in many situations. We ignore a third type of error: incorrectly assigning non-reference copy number states (e.g. calling a deletion as a duplication). Although we believe this type of error is non-trivial, particularly in CNV regions with high copy number, we feel that its impact on association testing should be minor, while modeling this error reduces the overall simplicity and generalizability of our results.

We define X_o_ as the observed integer copy number state, such as that called from a typical HMM CNV calling algorithm, given the true, underlying copy number state (X) and false positive and false negative error rates ν_p_ and ν_n_, respectively. Like X, X_o_ can take on integer values between zero and four. The joint probabilities of these variables are shown in Table S2. The observed copy number state can also be viewed as the realization of X after some error (Δ) is added, such that X_o_  =  X + Δ. Derivation of the moments of each variable are included in File S1. Variance displayed in CNV calling strictly due to errors is presented in Table 1 and Table 2 for select error rates and types of CNV loci (Table S3).

**Table 1:** Square root of the variance of Δ for the deletion and duplication CNV loci with and false negative (ν_n_) and false positive error rates (ν_p_).

		ν_p_		
		0	0.05	0.10
ν_n_	0	0	0.212	0.294
	0.2	0.206	0.301	0.368
	0.5	0.316	0.392	0.451
	0.7	0.367	0.438	0.495
	0.9	0.407	0.475	0.532

**Table 2:** Square root of the variance of Δ for the multiallelic CNV locus with and false negative (ν_n_) and false positive error rates (ν_p_).

		ν_p_		
		0	0.05	0.10
ν_n_	0	0	0.197	0.279
	0.2	0.283	0.345	0.397
	0.5	0.447	0.489	0.527
	0.7	0.529	0.565	0.598
	0.9	0.600	0.632	0.662

### Noise in Intensity Measurements

The log relative ratio (LRR) is defined as the logarithm with base 2 (log_2_) of the ratio between the overall allelic intensity at a given locus against the allelic intensity of some reference. In this manuscript we use the terms LRR and intensity measurements interchangeably. LRR values for a segment of DNA can be used to demonstrate the existence, boundary, and break points of a CNV. There are some issues with using LRR to assess association with CNVs. First, LRR is non-linear with respect to the underlying CNV. Without noise, a one copy number segment will have an LRR value of negative one, a two copy number segment will be zero, and a three copy number segment will be log_2_(3/2) ≈ 0.585 when using a two copy number segment as a reference. This will have a detrimental effect on association results when CNV is truly has an additive effect. Second, the LRR value for a zero copy number segment, in theory, should tend towards negative infinity. However, in practice some intensity is always observed due to background noise.

**Figure 1:**
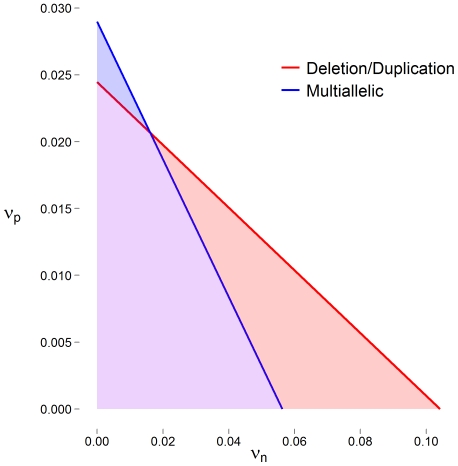
False positive (ν_p_) and false negative rates (ν_n_) for which the standard deviation of Δ is equal to 0.15 are represented as solid lines for a deletion-only or duplication-only CNV locus (red) or multiallelic locus, including both deletions and duplications (blue). Shaded areas represent sets of rates when the variance of Δ is less than the variance typically observed in LRR measurements. In these situations CNV calling algorithms are reducing measurement noise.

LRR values are never observed without some level of noise and uncertainty. If we define Z as the theoretical LRR given the underlying copy number state (Table S4) and Z_o_ as the observed LRR with added noise, then we can write Z_o_  =  Z + Δ_Z_, where Δ_Z_ represents the error in the LRR measurement induced by artifacts such as poor quality DNA and imprecise hybridization. Based on observation, standard deviation estimates of Δ_Z_ from array data are found to be near 0.15 for high quality DNA. Figure 1 displays ν_p_ and ν_n_ values for which Var(Δ)  =  Var(Δ_Z_)  =  0.15^2^. That is error rates for which the variance induced by errors in CNV calling algorithms is equivalent to the variance induced by noise in intensity measurements in typically observed array data.

### Power Simulation

We examined the impact that the inability to detect CNVs will have on association testing via simulation. A phenotype (Y) was simulated based on the model Y  =  Xβ + ε, where X is an indicator variable representing the presence or absence of a true, underlying CNV; β is the true effect of the CNV; and ε is error from a normal distribution with mean zero and variance σ^2^, denoted *n*[0, σ^2^]. The variance of Y was set to 100, β was set to 2.5, and the variance of ε was adjusted accordingly. Based on these parameters, a CNV present in 20% of the population would explain 1% of variance of the trait. We introduced a full range of false negative rates into the measurement of X, such that ν_n_ ∈ {0.00, 0.01, …, 0.99, 1.00}, and call this the CNV measurement observed after error, X_o_. We then regressed Y on X_o_.

**Figure 2:**
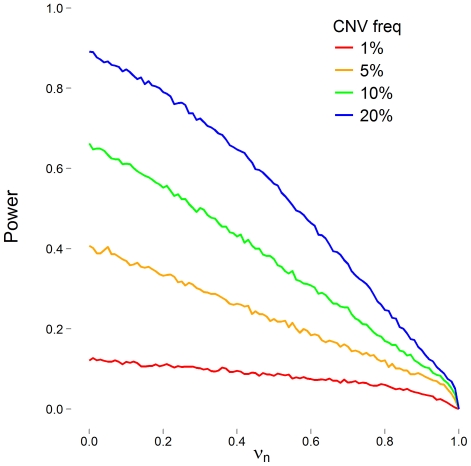
Simulated statistical power to detect an association with a putative CNV as a function of false negative rate (ν_n_). The CNV explains 1% of the phenotypic variation when present in 20% of the population. The CNV has a frequency of 1% (red), 5% (orange), 10% (green), or 20% (blue). False positive rate (ν_p_) is zero.

We considered true CNV frequencies of 1%, 5%, 10%, and 20%. For each set of CNV frequencies and false negative error rates, we performed 10,000 replicates in which phenotype, true CNV, and observed CNV were simulated for 1,000 subjects. We calculated power as the number of significant replicates divided by the total number of replicates. We set the significance level as α  =  0.05 and present results in Figure 2.

### Calling Algorithm Simulation

General trends concerning the relationship between CNV size, type, and recovery rates are known. For example, larger CNVs have a higher recovery rate than small CNVs, and deletions have a higher recovery rate than duplications. However, details as to when intensity measurements may be better suited in association analyses for certain sizes and types of CNVs are not. If a calling algorithm estimates copy number state without error, using CNV calls will be more powerful than intensity measurements which include noise. However, if a calling algorithm is producing many errors, such that the variance introduced by Δ is sufficiently large, then we expect the LRR to be more powerful to detect an association than the CNV calls. As the variance Δ is a function of error rates, we examined the scenarios in which one method is superior to the other, and vice versa.

**Figure 3:**
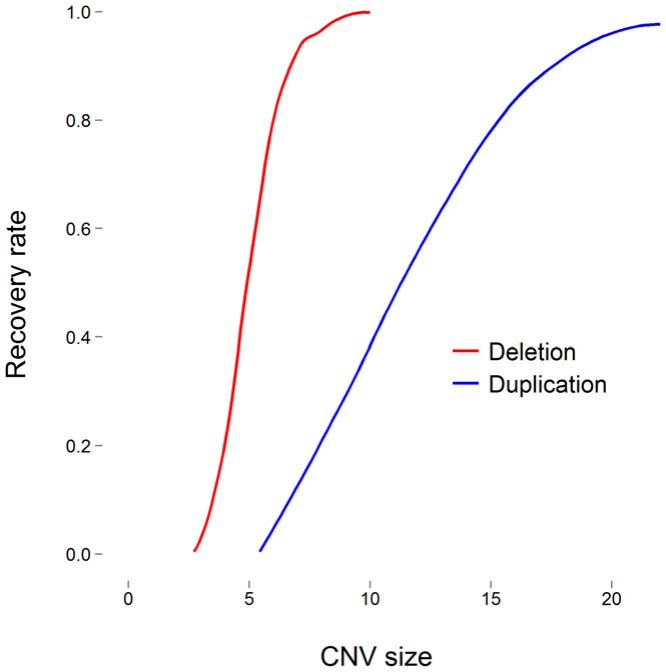
Recovery rate of deletions (red) and duplications (blue) from PennCNV using simulated intensity measurements as a function of CNV size.

To accomplish this, we first needed a detailed knowledge of the relationship between the size and type of CNV and the recovery rate. We simulated a region of DNA containing 10,000 CNV probes, each 1 kb apart, that was mostly devoid of CNV. In the center, we simulated a copy number variable region that ranged from 1 to 25 probes in length. We considered both single copy deletions and duplications. In each simulation, 200 subjects had a variant (deletion or duplication) in this region and 800 subjects did not. We simulated independent LRR values conditional on the underlying copy number state: non-variant and copy number invariable regions were simulated from a *n*[0, σ^2^] distribution; deletions from *n*[0.5 × (−1), σ^2^]; and duplications from *n*[0.5 × log_2_(3/2), σ^2^]. Mean values are one-half the theoretical values (Table S4) – a realization typically observed in array data. We set σ  =  0.15. We then used PennCNV [13] to call CNVs and observed recovery rates (Figure 3).

**Figure 4:**
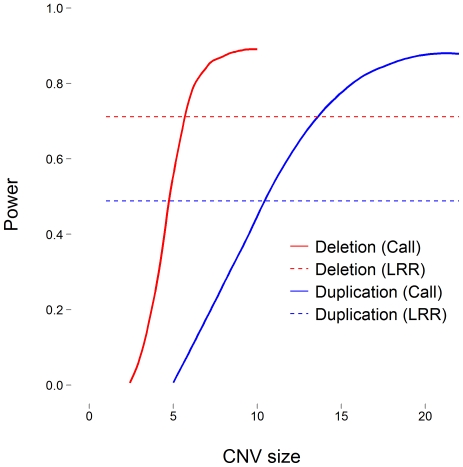
Statistical power to detect an association with a putative CNV as a function of CNV size. Red lines represent deletions and blue lines represent duplications. Solid lines represent power from calls made from CNV calling algorithms and dashed lines represent power from LRR.

Using the relationships between false negative error rates and power (Figure 2) and CNV size and recovery rate (Figure 3), we estimated the power of CNV calls to detect an association as a function of CNV size. We then calculated power using LRR at a single locus, as opposed to the CNV call, and present the results from both methods in Figure 4 for each type of CNV. In this example the CNV is present in 200 of 1,000 subjects and explains 1% of the phenotypic variance.

## Results

For even moderate error rates, the variance introduced in CNV calling due to errors exceeds the typical variance observed in intensity measurements (Table 1 and Table 2). In fact, only when error rates are very low, do we see smaller error variances (Figure 1). Not surprisingly, these errors cause a drop in the statistical power to detect association between a phenotype and putative CNV (Figure 2). The power loss appears to be somewhat uniform across CNVs of varying frequency. Compared to power without errors, we saw losses of 50% occurring between false negative rates of 0.55 and 0.65.

We found single copy deletions smaller than 4 probes and single copy duplications smaller than 5 probes to be virtually undetectable by PennCNV (Figure 3). The recovery rates improve as the size of the variant increases – more rapidly for deletions. Deletions larger than 7 probes have recovery rates greater than 90%. At 10 probes, nearly all the variants were detected. Alternatively, single copy duplications that were 10 probes long had a 39% recovery rate. The recovery rate of duplications did not exceeded 90% until the variant was 17 probes or larger.

Figure 4 displays the statistical power to detect an association between a phenotype and a putative CNV as a function of size. The power to detect an association increases for calls made by PennCNV as the CNV size (and recovery rate) increases. As deletions are less prone to error, they are more powerful than duplications at a given size. The tests achieve maximum power when there are no errors in the call. We discovered 80% power occurs for deletions larger than 6 probes and duplications larger than 15 probes. Meanwhile, power using LRR is represented as a dashed line. The calculation is invariant to the size of the CNV because we examined LRR associations at a single locus to avoid multiple testing issues and maintain comparability across methods. The power of LRR is 0.71 for deletions and 0.49 for duplications. Interesting points occur at the intersection of the methods for each type of variant. To the left, LRR is more powerful for smaller variants; and to the right, calls from CNV calling algorithms are more powerful for larger variants. We found LRR was more powerful for deletions smaller than 6 probes and duplications smaller than 11 probes.

## Discussion

We have shown that variance added to CNV genotyping calls due to errors in calling algorithms often exceeds the variance typically observed in LRR measurements. As error rates tend to be moderate to very high for many CNVs, the application of calling algorithms potentially creates additional, yet unseen variance. We have shown that this will have an impact on the power of association – explicitly showing this behavior as a function of error rates and CNV size. According to Zhang *et al*. (2011) [10], the lowest recovery rates occur for small CNVs, common CNVs, and duplications. Given that large losses in power occur with large error rates, it is not surprising that tests of association based on data from CNV calling algorithms have mostly identified large, rare deletions associated with disease risk and susceptibility. Perhaps only these types of CNVs have been sufficiently powered.

The results presented in this manuscript should be evaluated within the context of some limitations. Much of what we have shown is based on simulations performed under the assumptions that genetic data is consistent and follows a predictable pattern. While we do not feel that changes in our simulation parameters will have a drastic effect on the generalizability of our results, we do realize that the points and thresholds we noted with respect to recovery rate, power, and CNV size will likely be imprecise when applied other experimental conditions, including array-specific, locus-specific, and sample-specific differences. For these reasons, we tried to be conservative in our procedures.

If a CNV calling algorithm could entirely eliminate errors, then this method would be preferred. However, in the current environment that realization is not the case. Platforms for CNV genotyping and the calling algorithms themselves need to improve substantially. In the meantime, we suggest the LRR be used as the independent variable in association analyses when examining sufficiently small variants, or regions that appear to be invariant in copy number.

## Supporting Information

File S1 **Moments of the true CNV (X), the observed CNV (X_o_), and the error (Δ).**
(DOCX)

Table S1 **CNV genotype frequencies for given copy number states.**
(DOCX)

Table S2 **Joint probabilities of the true copy number state (X) and observed copy number state (X_o_), given CNV genotype frequencies, and false negative (ν_n_) and false positive error rates (ν_p_).**
(DOCX)

Table S3 **CNV genotype frequencies for each type of simulated CNV locus.**
(DOCX)

Table S4 **Theoretical values of LRR (Z), given the underlying copy number state X.**
(DOCX)
