# Supplementary material for: The Impact of Errors in Copy Number Variation Detection Algorithms on Association Results
Source: PLoS One. 2012 Apr 16;7(4):e32396. doi: 10.1371/journal.pone.0032396 (PMC3327691; doi:10.1371/journal.pone.0032396)
Supplement: Table S1 — CNV genotype frequencies for given copy number states. (DOCX) [file pone.0032396.s002.docx]

| Copy number | Genotype frequency |
| --- | --- |
| 0 | *f*_0_ |
| 1 | *f*_1_ |
| 2 | *f*_2_ |
| 3 | *f*_3_ |
| 4 | *f*_4_ |
