# Supplementary material for: The Impact of Errors in Copy Number Variation Detection Algorithms on Association Results
Source: PLoS One. 2012 Apr 16;7(4):e32396. doi: 10.1371/journal.pone.0032396 (PMC3327691; doi:10.1371/journal.pone.0032396)
Supplement: Table S2 — Joint probabilities of the true copy number state (X) and observed copy number state (Xo), given CNV genotype frequencies, and false negative (νn) and false positive error rates (νp). (DOCX) [file pone.0032396.s003.docx]

|  |  | X | | | | |  |
| --- | --- | --- | --- | --- | --- | --- | --- |
|  |  | 0 | 1 | 2 | 3 | 4 | |
| X_o_ | 0 | (1 – ν_n_)*f*_0_ | 0 | ν_p_*f*_0_*f*_2_/(1 – *f*_2_) | 0 | 0 | |
|  | 1 | 0 | (1 – ν_n_)*f*_1_ | ν_p_*f*_1_*f*_2_/(1 – *f*_2_) | 0 | 0 | |
|  | 2 | ν_n_*f*_0_ | ν_n_*f*_1_ | (1 – ν_p_)*f*_2_ | ν_n_*f*_3_ | ν_n_*f*_4_ | |
|  | 3 | 0 | 0 | ν_p_*f*_3_*f*_2_/(1 – *f*_2_) | (1 – ν_n_)*f*_3_ | 0 | |
|  | 4 | 0 | 0 | ν_p_*f*_4_*f*_2_/(1 – *f*_2_) | 0 | (1 – ν_n_)*f*_4_ | |
