# Supplementary material for: The Impact of Errors in Copy Number Variation Detection Algorithms on Association Results
Source: PLoS One. 2012 Apr 16;7(4):e32396. doi: 10.1371/journal.pone.0032396 (PMC3327691; doi:10.1371/journal.pone.0032396)
Supplement: Table S3 — CNV genotype frequencies for each type of simulated CNV locus. (DOCX) [file pone.0032396.s004.docx]

| Copy Number | Deletion | Duplication | Multiallelic |
| --- | --- | --- | --- |
| 0 | 0.01 | 0 | 0.01 |
| 1 | 0.18 | 0 | 0.16 |
| 2 | 0.81 | 0.81 | 0.66 |
| 3 | 0 | 0.18 | 0.16 |
| 4 | 0 | 0.01 | 0.01 |
