# Supplementary material for: The Impact of Errors in Copy Number Variation Detection Algorithms on Association Results
Source: PLoS One. 2012 Apr 16;7(4):e32396. doi: 10.1371/journal.pone.0032396 (PMC3327691; doi:10.1371/journal.pone.0032396)
Supplement: Table S4 — Theoretical values of LRR (Z), given the underlying copy number state X. (DOCX) [file pone.0032396.s005.docx]

| X | Z |
| --- | --- |
| 0 | -∞ |
| 1 | -1 |
| 2 | 0 |
| 3 | log_2_(3/2) |
| 4 | 1 |
