# Supplementary material for: The Impact of Errors in Copy Number Variation Detection Algorithms on Association Results
Source: PLoS One. 2012 Apr 16;7(4):e32396. doi: 10.1371/journal.pone.0032396 (PMC3327691; doi:10.1371/journal.pone.0032396)
Supplement: Supplementary file 5 [file pone.0032396.s001.docx]

$$E(\mathrm{X}) = \sum_{x=0}^{4} x \cdot f_x$$

$$E(\mathrm{X}^2) = \sum_{x=0}^{4} x^2 \cdot f_x$$

$$Var(\mathrm{X}) = E(\mathrm{X}^2) - \left[E(\mathrm{X})\right]^2$$

$$E(\mathrm{X}_o) = \sum_{x \neq 2} x \left[f_x (1 - \nu_n) + f_2 \cdot \nu_p \left(\frac{f_x}{1 - f_2} \right) \right] + 2\left[(1 - f_2)\nu_n + f_2 (1 - \nu_p) \right]$$

$$E(\mathrm{X}_o^2) = \sum_{x \neq 2} x^2 \left[f_x (1 - \nu_n) + f_2 \cdot \nu_p \left(\frac{f_x}{1 - f_2} \right) \right] + 4\left[(1 - f_2)\nu_n + f_2 (1 - \nu_p) \right]$$

$$Var(\mathrm{X}_o) = E(\mathrm{X}_o^2) - \left[E(\mathrm{X}_o)\right]^2$$

$$E(\mathrm{X}\mathrm{X}_o) = \sum_{x \neq 2} x \cdot f_x \left[x (1 - \nu_n) + 2\left(\nu_n + \nu_p \frac{f_2}{1 - f_2} \right) \right] + 4 \cdot f_2 (1 - \nu_p)$$

$$Cov(\mathrm{X}, \mathrm{X}_o) = E(\mathrm{X}\mathrm{X}_o) - \left[E(\mathrm{X}) \cdot E(\mathrm{X}_o) \right]$$

$$E(\Delta) = E(\mathrm{X}_o) - E(\mathrm{X})$$

$$Var(\Delta) = Var(\mathrm{X}) + Var(\mathrm{X}_o) - 2 \cdot Cov(\mathrm{X}, \mathrm{X}_o)$$
